# Cell proliferation and invasion is promoted by circSERPINA3 in nasopharyngeal carcinoma by regulating miR-944/MDM2 axis

**DOI:** 10.7150/jca.42799

**Published:** 2020-04-06

**Authors:** Rui Liu, Minghui Zhou, Puwen Zhang, Yulin Zhao, Yujie Zhang

**Affiliations:** Department of Rhinology, The First Affiliated Hospital of Zhengzhou University, Zhengzhou 450052, China

**Keywords:** circSERPINA3, miR-944, MDM2, nasopharyngeal carcinoma

## Abstract

Growing evidence has demonstrated that in tumor progression, circular RNAs (circRNAs) play important roles. However, the roles of circRNAs in nasopharyngeal carcinoma (NPC) have not been fully elucidated. In this study, it was demonstrated that that hsa_circ_0033074 (circSERPINA3) expression was found to be significantly upregulated in NPC tissues and cell lines. CircSERPINA3 inhibition significantly attenuated the invasion and proliferation abilities of NPC cells. The mechanism by which circSERPINA3 interacted with miR-944 was identified and MDM2 was demonstrated to function as a target gene of miR-944. Rescue experiments showed that miR-944 inhibitors or MDM2 overexpression reserved the effects of circSERPINA3 knockdown on NPC progression. Therefore, our study uncovered the circSERPINA3/miR-944/MDM2 axis in NPC, which may be a potential NPC therapeutic target.

## Introduction

Nasopharyngeal carcinoma (NPC) originates from the nasopharynx epithelium, which is a unique head and neck tumor 1, 2. In recent years, although great advancements have been achieved in fields of radiotherapy, adjuvant chemotherapy, and surgery an, the overall survival rate remains unsatisfied 3, 4. Therefore, the molecular mechanisms involved in NPC pathogenesis need to be explored.

Unlike linear RNAs, circular RNAs (circRNAs) have a characteristic covalent closed loop structure, with a lack of 5' 3' polarity or a polyadenylation tail 5, 6. A large number of studies have shown that circRNAs might play significant roles in certain biological processes, such as metastasis, apoptosis and proliferation 7, 8. As an example, Chen et al found that circPVT1 was found to serve as a prognostic marker and proliferative factor in gastric cancer progression by Chen et al. 9. circMTO1 was found to regulate the Wnt/β-catenin pathway, which resulted in a decrease colorectal cancer cell invasion and proliferation, in the study by Ge et al. 10. Chen et al. revealed that circRNA_100782 was able to regulate the proliferation of cells via the IL6-STAT3 axis in pancreatic carcinoma 11. However, the roles of circSERPINA3 in NPC development remain unclear.

In recent years, accumulating studies have illustrated that miR-944 may perform an important function for the progression of tumors. As an example, miR-944 was found to be able to suppress hepatocellular carcinoma progression through the deactivation of the PI3K/Akt axis and by regulating IGF-1R by Lv et al. 12. Pan et al. determined that miR-944 acts on the MACC1/Met/AKT axis to suppress gastric cancer metastasis via epithelial-mesenchymal transition inhibition 13. However, the involvement of miR-944 in NPC progression needs to be further elucidated.

This study confirmed that upregulated hsa_circ_0033074 (circSERPINA3) expression could promote NPC development. Subsequently, we demonstrated that circSERPINA3 executed its tumor oncogenic activity through sponging miR-944 and regulating the expression of MDM2. These results indicate that circSERPINA3 may function as an important target for NPC treatment.

## Materials and methods

### Samples of human tissue

30 primary NPC tissue samples and 30 non-tumor nasopharyngeal mucosa tissue (NT) samples were sourced from the First Affiliated Hospital of Zhengzhou University. Liquid nitrogen was used to snap-freeze the fresh tissues at -80℃ until use. Patients had not received any therapy before operation. The Ethics Committee of the First Affiliated Hospital of Zhengzhou University provided approval.

### Cell culture and transfection

Human nasopharyngeal epithelial cell line, NP-69, and the human NPC cell line (SNU46; SUNE1; CNE-1; 6‐10B; HNE-1; CNE-2 and HONE-1) were procured from Chinese Cell Bank of the Chinese Academy of Sciences. 10% fetal bovine serum (FBS; HyClone, USA), 100 U/ml penicillin and 100 μg/ml streptomycin (Gibco) supplemented Dulbecco's modified Eagle's medium (DMEM, Gibco, USA) was used to culture and maintain the cells at a temperature of 37°C in a wet 5% CO_2_ environment.

si-circSERPINA3 (si-circSERPINA3-1 sequence is 5′- ATTTCGTTGGAGTGGTCCATA-3′; si-circSERPINA3-2 sequence is 5′-AATTTCGTTGGAGTGGTCCAT-3′; si-circSERPINA3-3 sequence is 5′-GTTGGAGTGGTCCATAAGGCT-3′), miR-944 mimics, miR-944 inhibitors, MDM2 overexpression plasmid, and their negatively controls were created by GenePharma (China).

### Quantitative real‐time PCR (qRT‐PCR)

TRIzol reagents (Invitrogen) were used for RNA extraction from tissues and cells. A Prime Script RT Reagent Kit (Takara, Japan) and a One-step miRNA reverse transcription kit (Haigene, China) were used for reverse transcription. RT-qPCR was conducted utilizing the SYBR® Premix ExTaq (Takara) on a VII™7 System (Applied Biosystems). Expressions of circRNA, mRNA, and miRNA were calculated using the 2^-ΔΔCt^ method, and GAPDH or U6 was used as the internal reference. The primer sequences were: circSERPINA3, 5′- TGCAGAAAGGAGGGTGATTT-3′ (reverse) and 5′- GGCCTCCTGACAGCAATAAA-3′ (forward); U6, 5'‑ACGAATTTGCGTGTCATCCTTGCG‑3' (reverse) and 5'‑CTCGCTTCGGCAGCACATATACTA‑3' (forward); GAPDH, 5'‑TTGATTTTGGAGGGATCTCG‑3' (reverse) and 5'‑GAGTCAACGGATTTGGTCGT‑3' (forward).

### CCK8 assay

Cell Counting Kit-8 (CCK8; Roche, Switzerland) was used to determine cell proliferation. 10 μl of CCK8 solution was added into each well of 96-well plates containing 5 × 10^3^ cell. After 2 h, a microplate reader (Thermo scientific, USA) was used to measure absorbance at 450 nm.

### Colony formation assay

The cells were transferred into 6-well plates after transfection and were fixed using 4% paraformaldehyde for 10 min and 0.4% crystal violet was used to stain the cells, after 2 weeks. Finally, the number of visible colonies was determined.

### Transwell invasion assay

Serum-free media was used to transfect the cells. In order to determine cell invasion, the cells were added into the upper insert of a Transwell chamber (8 μm pore size, Millipore, Billerica, USA) pre-coated with Matrigel (Sigma, USA), while 10% FBS was added into the lower chamber. Methanol and 0.1% crystal violet were used to fix and stain the cells that had invaded after 24 h of incubation. A microscope (Olympus, Japan) was used to observe and count the cells.

### Dual-luciferase reporter assay

The mutant type luciferase reporter vectors (pmirGLO-circSERPINA3-Mut or pmirGLO-MDM2-Mut) and wide type luciferase reporter vectors (pmirGLO-circSERPINA3-WT or pmirGLO-MDM2-WT) were constructed by Genechem (Shanghai, China). Co-transfection of cells with miR-944 mimics (or miR-NC mimics) and luciferase reporter vectors were conducted separately. After 48 h, a dual-luciferase reporter assay system (Promegaand) was used to determine relative luciferase activity. Renilla luciferase activity was used for normalization.

### Statistical analysis

Statistical analyses were conducted using SPSS 22.0 software (USA). All data are presented as mean ± SD of experiments performed in triplicate. Student's t test or ANOVA were used to analyze differences. A* p* value of <0.05 was considered significant.

## Results

### circSERPINA3 was upregulated in NPC

**Figure 1:**
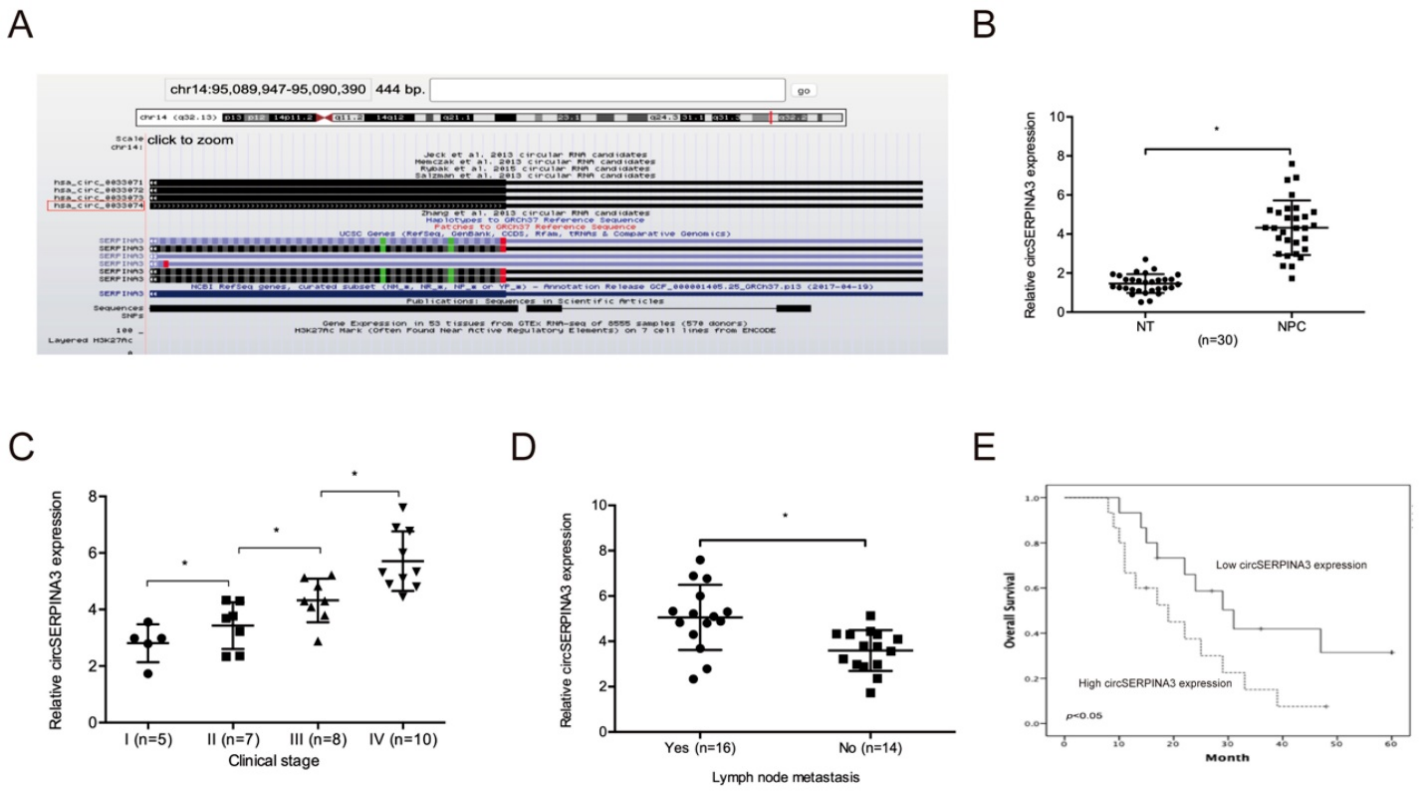
** circSERPINA3 expression in NPC.** (A) circSERPINA3 (hsa_circ_0033074) information. (B) circSERPINA3 was upregulated in NPC tissues. (C, D) High circSERPINA3 expression was associated with advanced clinical stage (C) and lymph-node metastasis (D) in NPC patients. (E) High circSERPINA3 expression in NPC patients was correlated with low overall survival rate. * p < 0.05.

Based on a previous study, we focused on a significantly upregulated circRNA, hsa_circ_0033074 (circSERPINA3) in HNSC 14. Hsa_circ_0033074 is spliced from the SERPINA3 gene, and the ultimate length is 443 nt (Figure 1A). Subsequently, we explored circSERPINA3 expression in NPC. QRT-PCR showed the significant upregulation of circSERPINA3 expression and its association with lymph-node metastasis and NPC patients of an advanced stage (Figure 1B-1D). Moreover, high expression of circSERPINA3 was found to be correlated with poor overall survival (OS) rate in NPC patients through the Kaplan-Meier analysis (Figure 1E).

### circSERPINA3 promoted NPC cell proliferation and invasion

**Figure 2:**
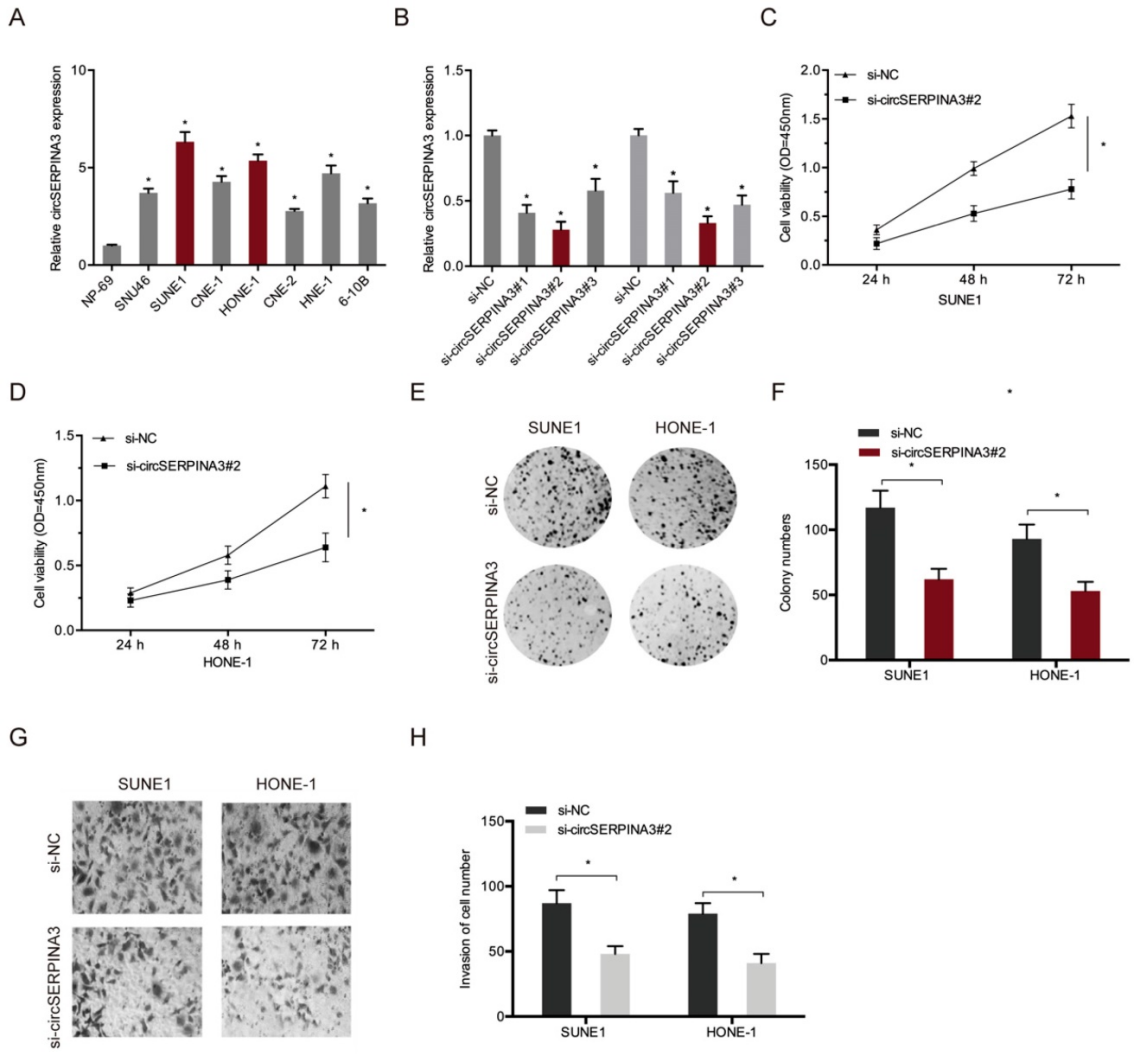
** circSERPINA3 accelerated the proliferation and invasion in NPC.** (A) circSERPINA3 was upregulated in NPC cell lines. (B) The efficiency of si-circSERPINA3 was detected by qRT-PCR. (C-F) Colony formation and CCK-8 assays were used to examine NPC cell proliferation abilities. (G, H) Transwell assay was used to explore NPC cells invasion abilities. * p < 0.05.

Next, we determined the function of circSERPINA3 in NPC progression. First, we analyzed the circSERPINA3 expression in NPC cell lines (Figure 2A). Next, si-circSERPINA3 and si-NC were transfected into SUNE1 and HONE-1 cells, and transfection efficiency was measured using qRT-PCR (Figure 2B). Colony formation and CCK-8 assays indicated that the downregulation of circSERPINA3 decreased SUNE1 and HONE-1 cell proliferation abilities *in vitro* (Figure 2C-2F). Transwell assay showed that circSERPINA3 inhibition decreased SUNE1 and HONE-1 cells invasion abilities in vitro (Figure 2G and 2H). Together, these results indicate that circSERPINA3 can function as an oncogeninc circRNA in NPC progression.

### circSERPINA3 interacted with miR-944

**Figure 3:**
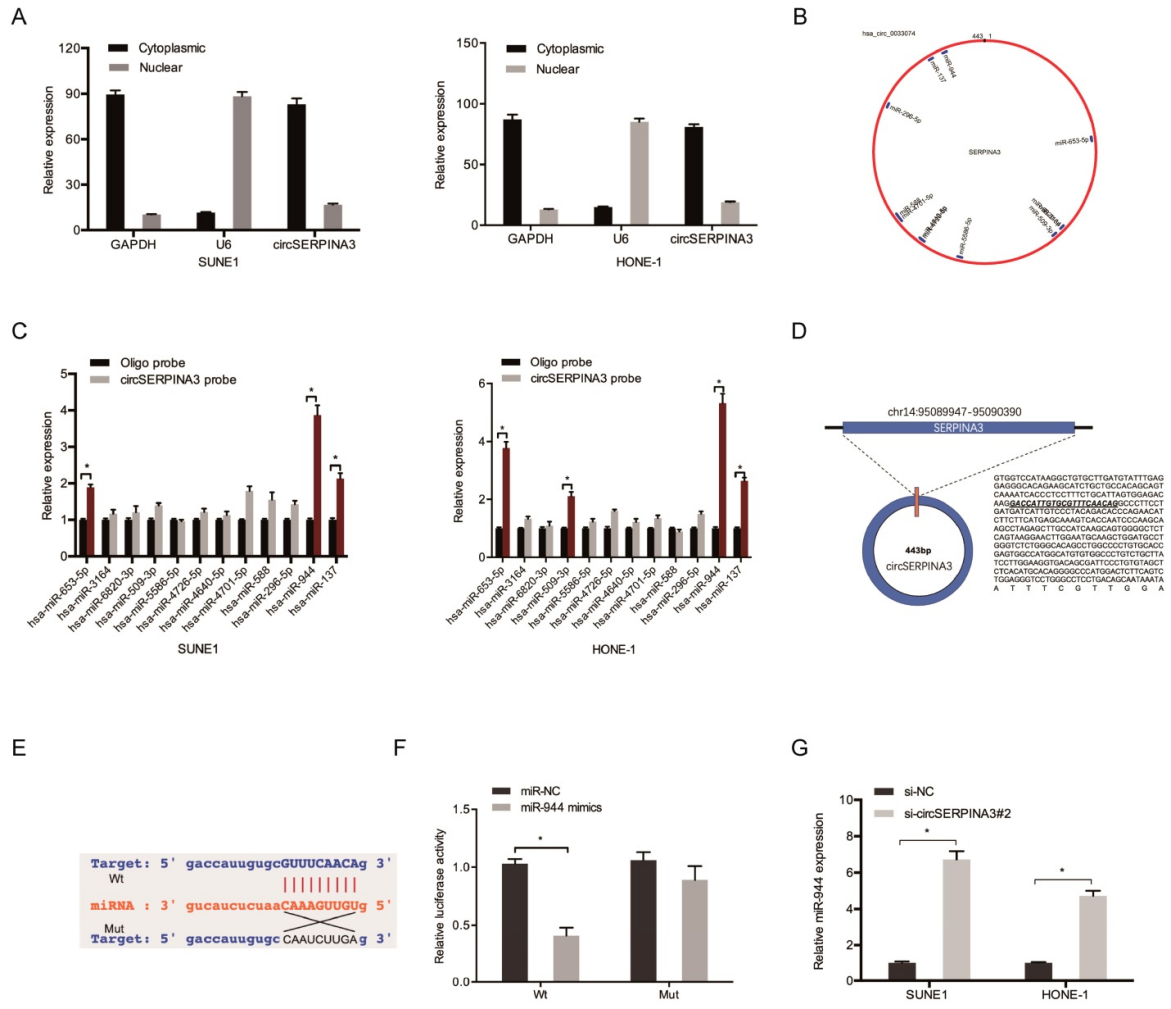
** circSERPINA3 interacted with miR-944.** (A) Subcellular fractionation assay demonstrated the scattering of circSERPINA3 in the cytoplasm. (B) Target miRNAs of circSERPINA3 was predicted by starBase. (C) The relative expression levels of 12 miRNA candidates in SUNE1 and HONE-1 cells lysates were detected by pull down assay. (D, E) The predicted miR-944 and circSERPINA3 binding sites. (F) MiR-944 mimics decreased the luciferase activity of the circSERPINA3-Wt group. (G) CircSERPINA3 inhibition increased miR-944 expression in NPC cells. * p < 0.05.

To further explore the underlying mechanism of circSERPINA3, subcellular fractionation assay was used. Results showed that circSERPINA3 was mostly scattered in cytoplasm (Figure 3A), indicating that circSERPINA3 might play regulatory roles in post-transcriptional level. Subsequently, we employed starBase to screen out 12 miRNAs which might probably bind to circSERPINA3 (Figure 3B). Biotin-labeled probe pull down assay showed that miR-944 was abundantly pulled down by circSERPINA3 probe in both SUNE1 and HONE-1 cells (Figure 3C). StarBase showed that circSERPINA3 was able to bind to miR-944 by binding sites (Figure 3D and 3E). miR-944 overexpression was found to have significantly decreased the luciferase activity of circSERPINA3-Wt, as observed using a luciferase reporter assay (Figure 3F). In addition, qRT-PCR was used to demonstrate that miR-944 expression in SUNE1 and HONE-1 cells increased as a result of circSERPINA3 inhibition (Figure 3G).

**Figure 4:**
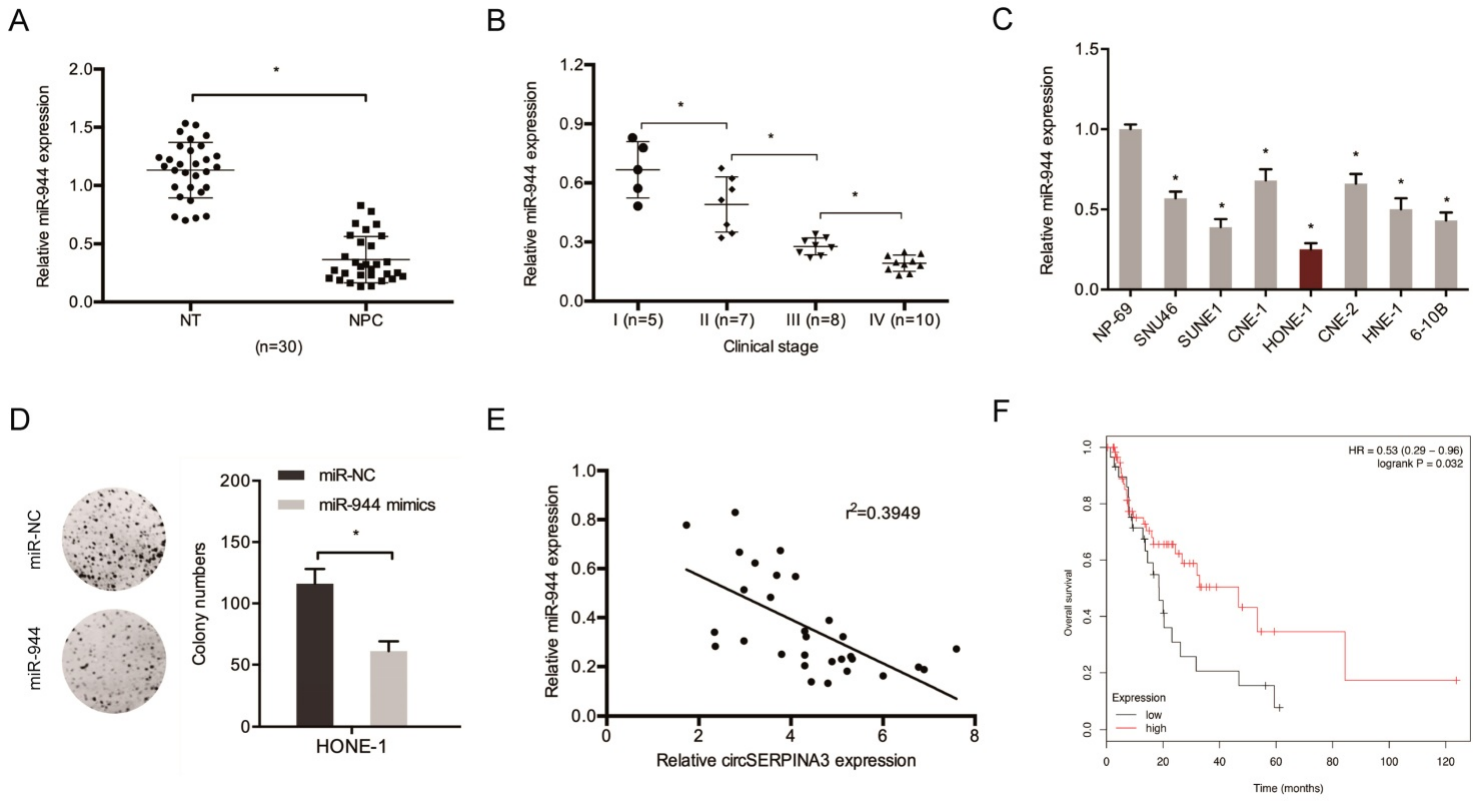
** miR-944 inhibited the proliferation in NPC.** (A) MIR-944 was downregulated in NPC tissues. (B) Low miR-944 expression in NPC patients was associated with advanced clinical stage. (C) MIR-944 was downregulated in NPC cell lines. (D) MiR-944 mimics reduced colony formation abilities in NPC cells. (E) The negative correlation between circSERPINA3 expression and miR-944 expression in NPC tissues. (F) Low miR-944 expression in NPC patients was associated with poor overall survival rate. * p < 0.05.

Next, miR-944 expression in NPC tissues was determined. The results showed the significant downregulation of the expression of miR-944 in NPC cell lines and tissues (Figure 4A and 4C). An association was found between low miR-944 expression and an advanced clinical stage among NPC patients (Figure 4B). Colony formation assay demonstrated that miR-944 mimics could decrease HONE-1 cell proliferation *in vitro* (Figure 4D). circSERPINA3 expression showed a negative correlation with miR-944 expression in NPC tissues, as shown through the correlation analysis (Figure 4E). In addition, low miR-944 expression was found to be related with poor overall survival of NPC patients, as shown by the Kaplan-Meier analysis (Figure 4F).

### MDM2 is a target gene of miR-944

**Figure 5:**
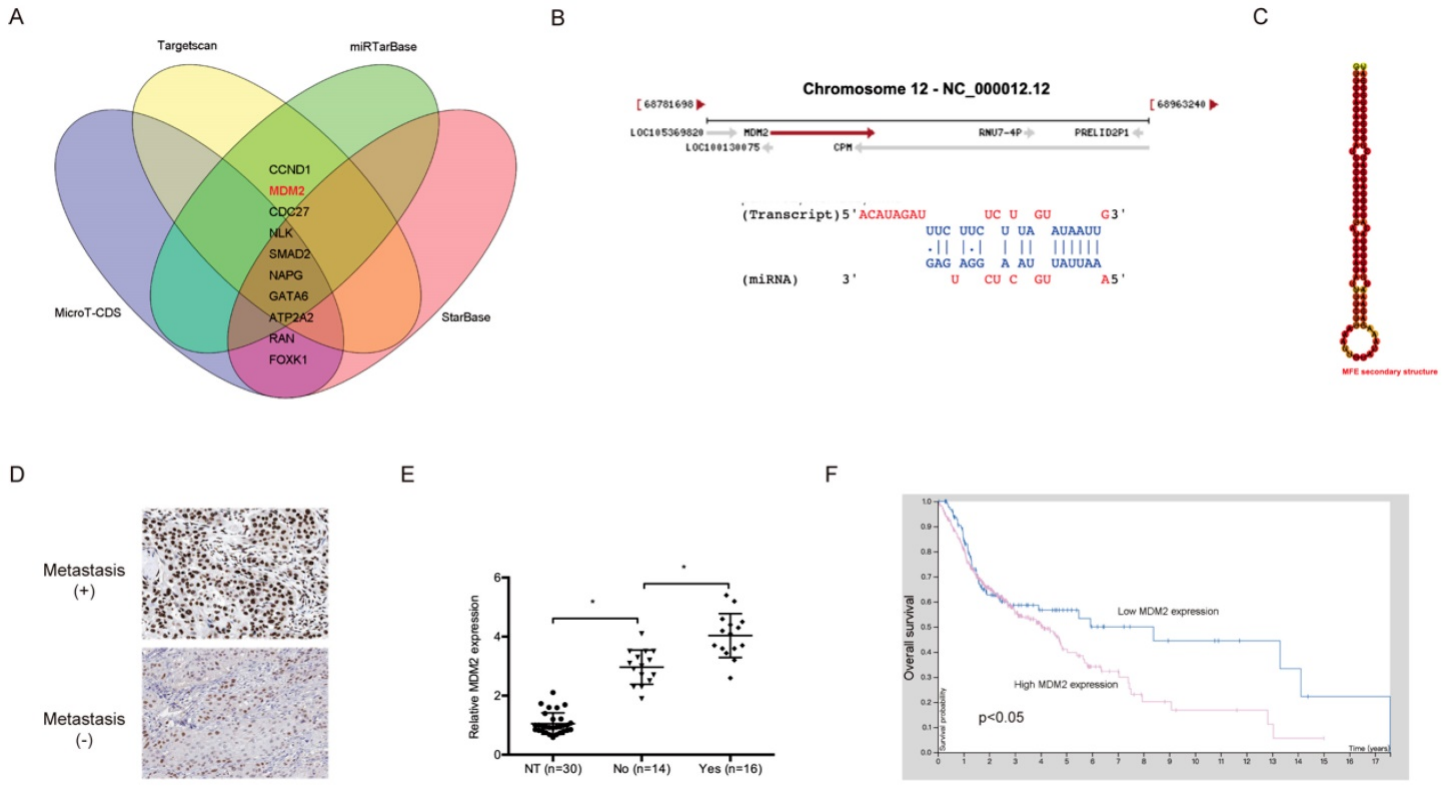
** MDM2 was a target gene for miR-944.** (A) Venn diagram exhibited the target genes of miR-944. (B) The sites of binding between miR-944 and MDM2. (C) The second structure of miR-944. (D) High MDM2 expression in NPC patients was associated with metastasis. (E) MDM2 was upregulated in NPC tissues. (F) High MDM2 expression in NPC patients was associated with poor overall survival rate. * *p* < 0.05.

The downstream target genes of miR-944 were further explored. The results show that MDM2 is a highly putative target gene of miR-944 (Figure 5A-5C). Subsequently, IHC showed that MDM2 was significantly upregulated in NPC patients with metastasis (Figure 5D), which was confirmed using qRT-PCR (Figure 5E). In addition, an association between high MDM2 expression and poor overall survival of NPC patients was found through Kaplan-Meier analysis (Figure 5F).

**Figure 6:**
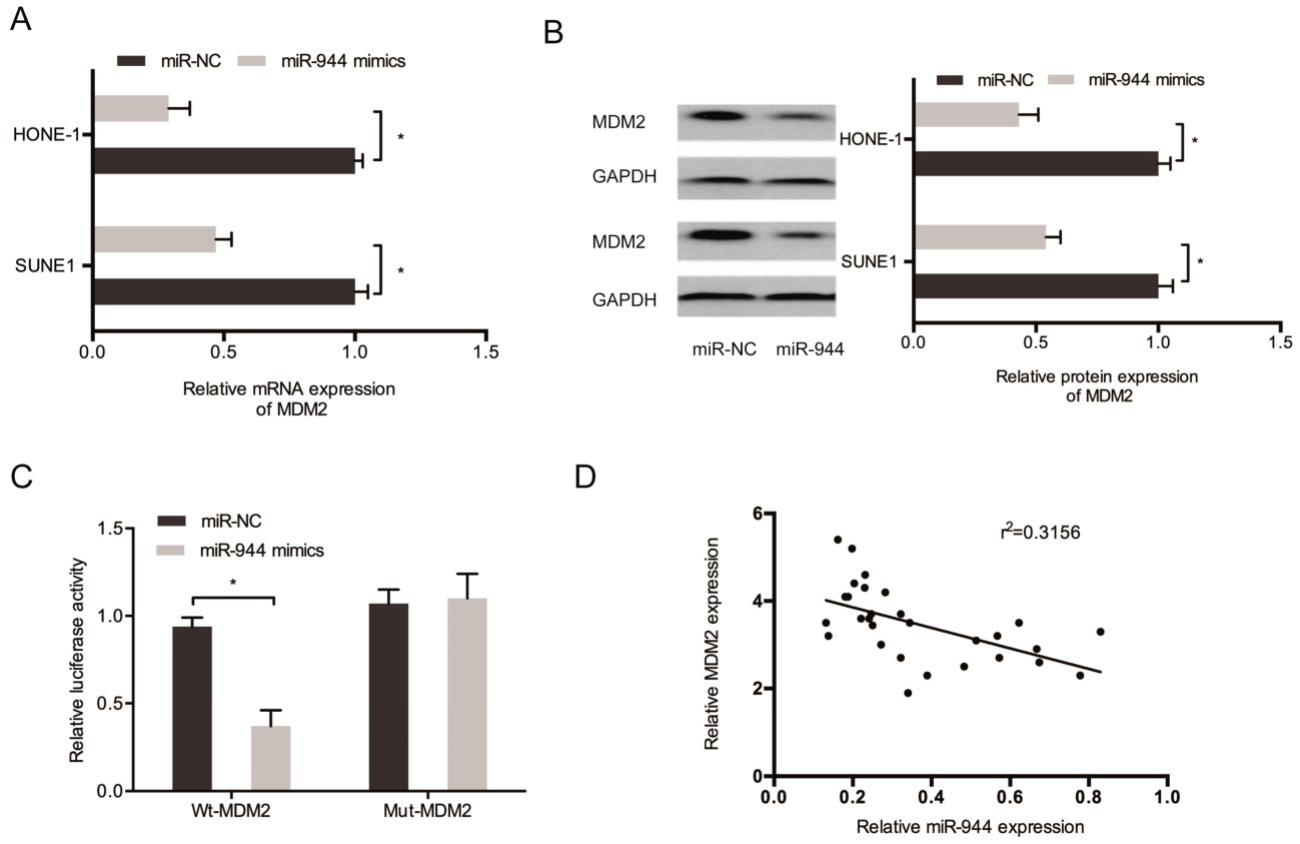
** MDM2 was a target gene for miR-944.** (A, B) MiR-944 mimics reduced MDM2 mRNA and protein levels in NPC cells. (C) MiR-944 mimics attenuated the luciferase activity of MDM2-Wt group. (D) MiR-944 expression was negatively correlated with MDM2 expression in NPC tissues. *p < 0.05.

Then, we aimed to determine the regulation by miR-944 on MDM2 expression. Results showed the significant decrease of protein and mRNA levels of MDM2 in SUNE1 and miR-944 mimic-transfected HONE-1 cells (Figure 6A and 6B). miR-944 mimics caused a decrease in the luciferase activity of MDM2-Wt group, but not the MDM2-Mut group, as observed using Kaplan-Meier analysis (Figure 6C). In addition, a negative correlation was found between miR-944 and MDM2 expression in NPC tissues (Figure 6D). These results confirmed that MDM2 was targeted by miR-944 in NPC.

### circSERPINA3 regulated NPC progression through the regulation of the miR-944/MDM2 axis

**Figure 7:**
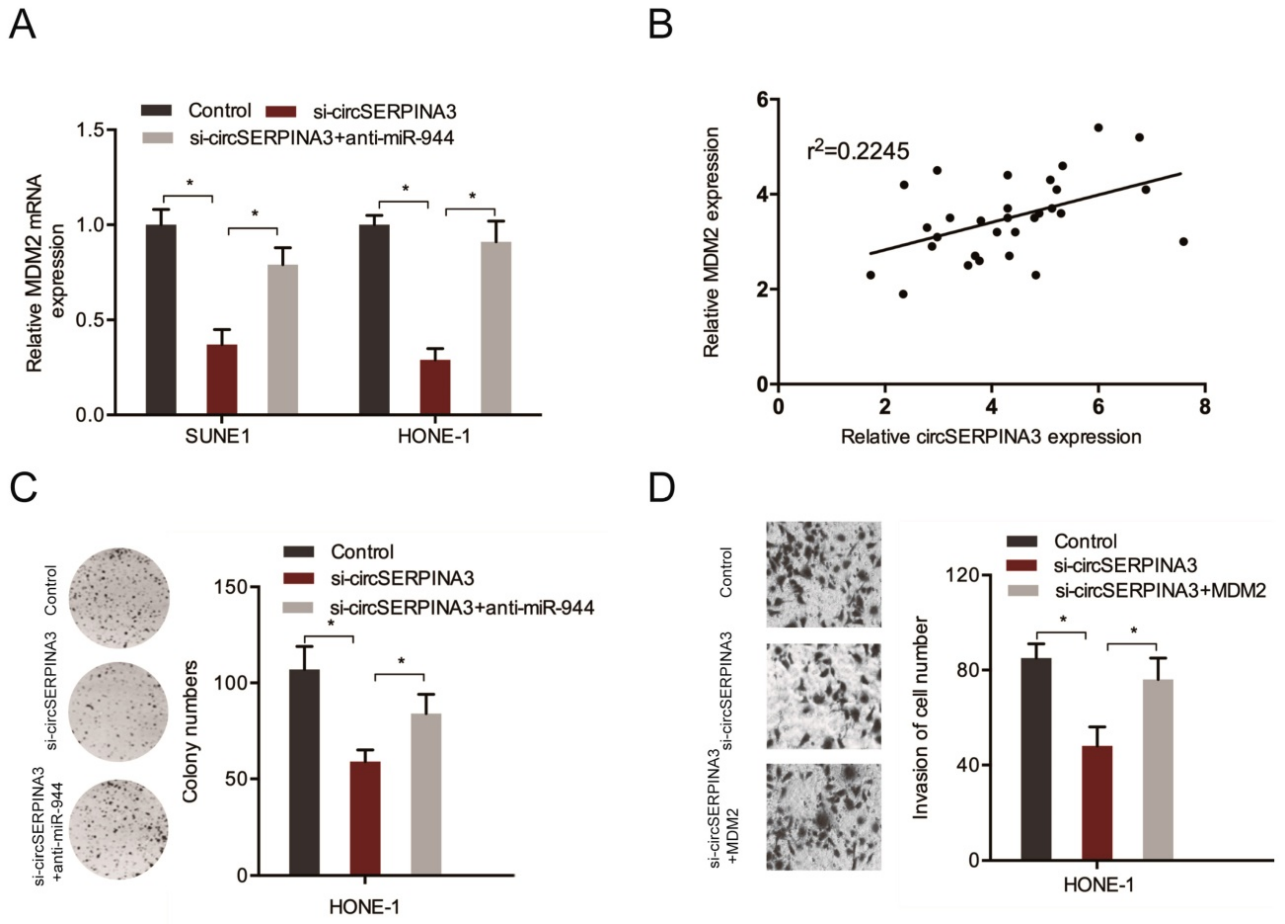
** circSERPINA3/miR-944/MDM2 axis in NPC.** (A) MiR-944 inhibitors reversed the effects of si-circSERPINA3 on MDM2 expression. (B) The positive correlation between circSERPINA3 expression and MDM2 expression in NPC tissues. (C) MiR-944 inhibitors reversed the proliferation effects of si-circSERPINA3 on HONE-1 cells. (D) MDM2 overexpression completely halted the effects of si-circSERPINA3 on HONE-1 cells invasion. *p < 0.05.

In order to further confirm the circSERPINA3/miR-944/MDM2 axis, the expression of MDM2 in SUNE1 and HONE-1 cells transfected with si-circSERPINA3 and miR-944 inhibitors were determined. Downregulated levels of circSERPINA3 were found to result in a significant decrease in MDM2 expression, while miR-944 inhibitors reversed the effects (Figure 7A). Subsequently, a positive correlation was found between circSERPINA3 and MDM2 expression in NPC tissues (Figure 7B), while the axis was further confirmed in NPC using rescue assays. Colony formation assays were used to demonstrate that the silencing of circSERPINA3 decreased NPC cell proliferation, while miR-944 inhibitors abolished the roles (Figure 7C). Transwell assay revealed that the cell invasion capacities were reduced after circSERPINA3 interference, whereas the decreased invasion capacity was restored by MDM2 overexpression (Figure 7D). Therefore, we suggested that regulation of the miR-944/MDM2 axis by circSERPINA3 could promote NPC progression.

## Discussion

In recent times, a number of studies have shown that circRNAs perform critical functions in NPC carcinogenesis. As an example, in NPC patients, Shuai et al. found an association between circRNA_0000285 overexpression and advanced clinical features of NPC patients 15. Ke et al. found that CircHIPK3 promoted NPC cell invasion and proliferation through targeting the miR-4288/ELF3 axis 16. Zhong et al. demonstrated that cRNA CDR1 promotes NPC cell invasion and proliferation by regulating the miR-7-5p/E2F3 axis 17. However, the functions as well as underlying mechanisms of circRNAs in NPC development are still not well known.

In this study, circSERPINA3 was identified as a key upregulated circRNA in NPC development. High circSERPINA3 expression in NPC patients was associated with advanced clinical stage, lymph node metastasis, and poor overall survival rate. Subsequently, loss-of-function experiments showed that circSERPINA3 might exert oncogenic functions by aggravating cell invasion and proliferation abilities in NPC progression.

Recently, increasing evidence showed that circRNAs could function as a competing endogenous RNAs (ceRNAs) for the progression of various tumors, including NPC 18. As an example, Wei et al. determined that circ_0008450 increased CXCL9 expression by regulating miR-577 to regulate NPC cells proliferation and invasion 19. Zhu et al indicated that circRNA ZNF609 functioned as an oncogene in NPC progression to regulate miR-150-5p 20. In our study, we showed circSERPINA3 was primarily found in the cytoplasm, indicating that circSERPINA3 may use the ceRNA network to regulate NPC progression. The bioinformatics analysis showed that circSERPINA3 contains a mR-944 binding site. Thereafter, the interaction between miR-944 and circSERPINA3 in NPC cells was confirmed using dual luciferase assays and qRT-PCR. Moreover, rescue experiments showed that miR-944 reversed the effects of circSERPINA3 on NPC cells. Thus, we indicated that circSERPINA3 may function by sponging miR-944.

The tumor suppressor p53 plays a central role in tumor prevention 21. E3 ubiquitin ligase MDM2 is the most critical negative regulator for p53. MDM2 binds to p53 and ubiquitinates p53 for proteasomal degradation 22, 23. Recently increasing studies showed that MDM2 performs important functions in tumor progression. As an example, Chen et al found that MDM2 promoted epithelial-mesenchymal transition and metastasis in ovarian cancer 24. Jiang et al. demonstrated that miR-758-3p suppressed hepatocellular carcinoma cells proliferation, and invasion by targeting MDM2 and mTOR 25. Li et al found that HBXIP promoted tumor growth via down-regulating p53 via miR-18b/MDM2 and pAKT/MDM2 pathways in breast cancer 26. In this study, MDM2 was found to be a downstream gene of miR-944. MDM2 expression was positively mediated by circSERPINA3 and reservedly modulated by miR-944. Rescue assays showed that MDM2 overexpression abolished the roles of circSERPINA3 deficiency on NPC cells invasion.

## Conclusions

In conclusion, we revealed the significant upregulation of circSERPINA3 expression in NPC and demonstrated its correlation with advanced clinical features, while circSERPINA3 enhanced NPC progression by targeting the miR-944/MDM2 axis. Thus, we suggested that circSERPINA3 might serve as an effective therapeutic in NPC treatment.

## Abbreviations

NPC: Nasopharyngeal carcinoma
circRNA: Circular RNAs
miRNAs: MicroRNAs
Mut: Mutant
WT: Wild type
UTR: Untranslated region
